# Fibrinogen in mice cerebral microvessels induces blood–brain barrier dysregulation with aging via a dynamin-related protein 1–dependent pathway

**DOI:** 10.1007/s11357-023-00988-y

**Published:** 2023-10-28

**Authors:** Partha K. Chandra, Manesh Kumar Panner Selvam, Jorge A. Castorena-Gonzalez, Ibolya Rutkai, Suresh C. Sikka, Ricardo Mostany, David W. Busija

**Affiliations:** 1 Department of Pharmacology, Tulane University School of Medicine, 1430 Tulane Avenue, New Orleans, LA 70112 USA; 2 Tulane Brain Institute, Tulane University, 200 Flower Hall, 6823 St. Charles Avenue, New Orleans, LA 70118 USA; 3 Department of Urology, Tulane University School of Medicine, 1430 Tulane Avenue, New Orleans, LA 70112 USA

**Keywords:** Brain aging, Cortical microvessels, Proteomics, Protein–protein interaction network, Mitochondrial fission/fusion, Endothelial/blood–brain barrier dysfunction

## Abstract

We previously reported evidence that oxidative stress during aging leads to adverse protein profile changes of brain cortical microvessels (MVs: end arterioles, capillaries, and venules) that affect mRNA/protein stability, basement membrane integrity, and ATP synthesis capacity in mice. As an extension of our previous study, we also found that proteins which comprise the blood–brain barrier (BBB) and regulate mitochondrial quality control were also significantly decreased in the mice’s cortical MVs with aging. Interestingly, the neuroinflammatory protein fibrinogen (Fgn) was increased in mice brain MVs, which corresponds with clinical reports indicating that the plasma Fgn concentration increased progressively with aging. In this study, protein–protein interaction network analysis indicated that high expression of Fgn is linked with downregulated expression of both BBB- and mitochondrial fission/fusion–related proteins in mice cortical MVs with aging. To investigate the mechanism of Fgn action, we observed that 2 mg/mL or higher concentration of human plasma Fgn changed cell morphology, induced cytotoxicity, and increased BBB permeability in primary human brain microvascular endothelial cells (HBMECs). The BBB tight junction proteins were significantly decreased with increasing concentration of human plasma Fgn in primary HBMECs. Similarly, the expression of phosphorylated dynamin-related protein 1 (pDRP1) and other mitochondrial fission/fusion–related proteins were also significantly reduced in Fgn-treated HBMECs. Interestingly, DRP1 knockdown by shRNA(h) resulted in the reduction of both BBB- and mitochondrial fission/fusion–related proteins in HBMECs. Our results suggest that elevated Fgn downregulates DRP1, leading to mitochondrial-dependent endothelial and BBB dysfunction in the brain microvasculature.

**Supplementary information:**

The online version contains supplementary material available at 10.1007/s11357-023-00988-y.

## Introduction

Aging is an inevitable stress with escalating deleterious effects on brain microvessels (MVs: end arterioles, capillaries, and venules), which influences neuronal health and function and increases susceptibility to neurocognitive impairment and dementia. Healthy cerebral MVs are critical for the maintenance of nutrient supply for brain metabolic requirements while providing immunological and physical protection of brain tissues from damaging substances via the blood–brain barrier (BBB). Age-related anatomical changes on cerebral MVs have been reported and include decreases in small blood vessels and capillary density [1–4], looping, tortuosity, twisting, reorganization [5–10], and BBB leakage [11, 12]. Research on the role of the various proteins in cerebral MVs in health and disease during aging has been relatively neglected due to the previous focus on large arteries and the methodological challenges in understanding the complex factors involved in the synthesis, stability, function of proteins, and protein–protein interactions.

We recently examined the expression and interactions of large numbers of differentially expressed proteins (DEPs) in mouse brain MVs and reported that reduced ROS scavenging ability and mRNA/protein stability are likely early precipitating events leading to the adverse protein changes that reduce ATP production by glycolysis and mitochondrial respiration and compromise the structural integrity of the BBB in mice cortical MVs with aging [13]. An unexpected finding was that the fibrinogen (Fgn) content of brain MVs increases with aging. However, the impact of Fgn on the brain microvasculature with aging and on energy production and BBB status has not been examined. In the current study, we performed a more extensive examination of protein–protein interaction networks, including those involved in the maintenance of BBB integrity and mitochondrial fission/fusion programming in cortical MVs of young, middle-aged, and old mice. Specifically, we focused on the initiating pathogenic role of Fgn in MV disfunction with aging.

## Materials and methods

### Animals

Young (4–6 months), middle-aged (12–14 months), and old (20–21 months) mice were incorporated in this study. Tg(Thy1-EGFP)MJrs/J] (Jax No. 007788) mice (Jackson Laboratory) were bred in a C57B16J background [13]. We included six mice (*n* = 6) in each group. An equal number of age-matched male and female mice were included in each group. Mice were maintained in group housing at ~ 23°C on a 12-h light/dark cycle with ad libitum access to food and water. This study followed the Institutional Animal Care and Use Committee guidelines of Tulane University, the National Institutes of Health Office of Laboratory Animal Welfare guidelines, and the ARRIVE guidelines for animal research. To avoid any differences due to circadian rhythm, cortical MVs were collected at the same time of day from all mice.

### Microvessels isolation

The MV isolation protocol was described previously [13–15]. Briefly, euthanasia was done using isoflurane anesthesia followed by decapitation. The mouse brain was removed and placed on filter paper and the hindbrain, olfactory bulbs, white matter, and large surface blood vessels were removed and discarded. The remaining cortical tissue was homogenized with 5 mL ice-cold Dulbecco’s phosphate-buffered saline (DPBS) (Life Technologies Corporation, NY, USA) on ice, and centrifuged at 3300 × g for 15 min. The pellet was resuspended in 17.5% dextran (Therma Fisher Scientific, Waltham, MA), and filtered through a 300 µm filter unit (pluriSelect Life Science, CA, USA). The filtrate was centrifuged at 7900 × g for 15 min. The MV pellet was resuspended in 2% bovine serum albumin (BSA) (Sigma-Aldrich, St Louis, MO), and passed through a 70 µm filter (Corning Incorporated, NY, USA). To get contamination-free MVs, the subsequent sample was centrifuged at 13,000 × g for 15 min with a final clean-up with 17.5% dextran followed by 2% BSA. Finally, the MV pellet was resuspended in PBS, its integrity was validated as described in our studies [13–17] and stored at − 80 °C until used.

### Sample preparation for proteomic analysis

Samples were prepared for proteomic analysis by following our published protocol [14]. Briefly, PBS was removed by centrifugation (10,000 rpm/5 min), MV pellets were lysed in 1% SDS solution with intermediate vortexing and sonication. Protein quantitation was performed using bicinchoninic acid (BCA) Protein Assay Kit (Thermo Scientific, Rockford, IL) and 100 µg of each sample was used for TMT labeling.

### Quantitative discovery-based proteomic analysis in mice cortical MVs

Detailed procedures were described in our previous publications [13–15]. Briefly, 100 µg of each protein sample was prepared for trypsin digestion followed by alkylation with iodoacetamide. After chloroform–methanol precipitation, each protein pellet was digested with 1 µg trypsin overnight at 37 °C. Tryptic peptides were labeled using one of the three tandem mass tags (TMT) 6-plex reagent sets (Thermo Scientific Pierce). An equal amount of each TMT-labeled sample was combined in a single tube with SepPak purified (Waters, Ireland) using acidic reverse-phase conditions. The fractionated, labeled peptide mixtures were run on a Dionex U3000 nano-flow system (Thermo Fisher Fusion Orbitrap mass spectrometer). Chromatography was conducted in a “trap-and-load” format using an EASY-Spray source. The entire run had a flow rate of 0.3 µL/min and electrospray was achieved at 1.8 kV. The 3 runs of each age group were searched using the SEQUEST HT node of Proteome Discoverer 2.4 (Thermo Scientific). The Protein FASTA database was the *Mus musculus*, SwissProt tax ID = 10′090, version 2017–10-25 containing 25,097 sequences. Using a false discovery rate (FDR) of < 1%, only one unique high-scoring peptide of an identified protein was needed for addition in our results. Proteome Discoverer was also used to determine the quantitative differences between biological groups.

### Bioinformatic analysis

The effects of aging on the proteome were determined by comparing the differentially expressed proteins (DEPs) of young, middle-aged, and old mice. Proteins identified in all three study groups were uploaded to ingenuity pathway analysis (IPA) software. Initially, core analysis was conducted, and then, interaction network analysis was performed to identify the interaction between Fgn and proteins involved in BBB and the tight junction (TJ)-signaling pathway as well as the mitochondrial fission/fusion process. Protein–protein interaction was demonstrated based on the following criteria: experimental evidence, neighborhood, gene fusion, occurrence, co-expression, existing databases, and text mining. Comparisons were also carried out between the three analyzed datasets (old vs. young, middle-aged vs. young, and old vs. middle-aged) to determine the differences in the identity of the top diseases and biological functions regulated by the DEPs in mice cortical MVs with aging.

### Cell culture

Primary human brain microvascular endothelial cells (HBMECs) (Catalog # ACBRI 376), culture media, and reagents were purchased from the Cell System and cultured as shown previously [18, 19]. Briefly, cells were cultured in a complete medium containing 10% fetal bovine serum and other reagents according to the manufacturer’s recommendations. Cells were washed with passage reagent group (PRG)-1, dissociated with PRG-2, and the enzymatic reaction stopped with ice-cold PRG-3. Cells were centrifuged (200 × g for 7 min at 4 °C), resuspended in the complete classic medium, seeded in flasks coated with attachment factor (4Z0-210), and incubated at 37 °C with 5% CO_2_ in 95% relative humidity. Cells were cultured with fresh media every 48 h and were used up to passage 9 in different experiments.

### Reagents and antibodies

Fibrinogen from human plasma (#F3879) and 3-(4,5-dimethylthiazol-2-yl)-2,5-diphenyl tetrazolium bromide (MTT) reagent (#M5655) were purchased from Sigma-Aldrich, St. Louis, MO. DRP1 shRNA(h) lentiviral particles (#sc-43732-V), Control shRNA lentiviral particles-A (#sc-108080), and Polybrene® (#sc-134220) were purchased from Santa Cruz Biotechnology, Dallas, TX, USA. Antibodies were purchased from the following suppliers: against ZO-2 (#2847), phospho-DRP1[S616] (#3455), phospho-DRP1[S636] (#4867), and claudin-5 (#49,564) from Cell Signaling Technology, Danvers MA, USA; against JAM-A (#sc-53623) and occludin (#sc-133256) from Santa Cruz Biotechnology, Dallas, TX, USA; against β-actin (#A5441), and MFN2 (#M6444) from Sigma-Aldrich, St. Louis, MO, USA; against total DRP1 (#611,112) and OPA1 (#612,606) from BD Transduction Laboratory, San Jose, CA, USA; against PECAM-1 (#01004) from BiCell Scientific, Maryland Heights, MO, USA; against FIS1 (#ALX-210–1037) from Enzo Life Sciences, Inc, Farmingdale, NY, USA; and against VE-cadherin (#36–1900) from Invitrogen, Frederick, MD, USA.

### Quantitative measurement of cell morphology

Images of cultured HBMECs were collected under control conditions and following treatment with different concentrations of Fgn. Pictures were taken with bright field under light microscopy and representative pictures of the treated or untreated (mock) cells were compared for change in morphology. To quantitatively assess the effects of fibrinogen treatment on the cell morphology, we measured the aspect ratio and the circularity of cells using ImageJ Fiji. Briefly, images were converted from RGB to 16-bit grayscale and then subjected to automated contrast enhancement. In each field of view, 10–20 cells were manually segmented using the freehand selection tool. Segmented cells were then converted to a black-and-white mask containing only these selected objects. Using the analyze particles tool, objects were automatically detected, and their morphology was characterized by measuring various shape descriptors, including aspect ratio and circularity. Aspect ratio refers to the ratio of the major to minor axis from an elliptical fit to the cell shape, whereas circularity is a parameter that ranges from 0 to 1, with 1 being a perfect circle. Therefore, cells undergoing elongation will display higher aspect ratio values and lower circularity values. Statistical difference was determined using one-way ANOVA followed by the Tukey test for multiple comparisons.

### Measurement of cell viability

The viability of primary HBMECs after the treatment with human Fgn for 24 h was assessed by MTT assay. Briefly, 5000 cells were seeded in 96-well plates with 200 µL complete classic media. The next day, cells were cultured with fresh media and treated with different concentrations (1–8 mg/mL) of human Fgn. The cell viability was measured at 24 h post-exposure to Fgn with MTT solution (5 mg/mL for 3–4 h at 37 °C). DMSO was used to solubilize the formazan crystals, and the optical density was measured at 540 nm with a spectrophotometer (BioTek Instruments).

### Transwell-based BBB permeability assay

Following the published protocol [18, 19], primary HBMECs were seeded onto transwell inserts and cells were allowed to grow for 3 days in a complete classic medium. After cells became confluent on the transwell, HBMECs were treated with human plasma–derived Fgn in fresh culture medium for 24 h. The cell monolayer’s ability to limit the infiltration of a medium to high molecular weight (70 and 150 kDa) fluorescein isothiocyanate-labeled dextran (FITC-dextran; Sigma-Aldrich, St. Louis, MO, USA) was determined before and after the addition of 10 µg/mL FITC-dextran to the upper transwell chamber. After incubation for 30 min, 20 µL of medium was collected from the lower chamber, and the fluorescence was measured with a spectrophotometer (BioTek Instruments, Winooski, VT, USA) set to 485/20 nm excitation and 528/20 nm emission.

### Western blotting

We followed our previously published laboratory protocol to prepare the cell lysates and perform immunoblots [14, 15, 19, 20]. In brief, NP40 lysis buffer (Invitrogen, Frederick, MD, USA) with phosphatase and protease inhibitors was used to lyse the cells, and the protein concentration of the clarified lysates was measured by Pierce BCA protein assay (Thermo Scientific). Proteins were separated using a gradient gel (4–20% SDS-PAGE) and transferred onto a PVDF membrane. A 1X blocking buffer (Abcam, Cambridge, MA, USA) was employed to block the non-specific binding sites and to dilute the primary antibodies. The membranes were rinsed with Tris-buffered saline (Bio-Rad, Hercules, CA, USA) with 0.1% Tween-20 (Sigma-Aldrich, St. Louis, MO, USA), and incubated overnight with primary antibodies at 4 °C. The next day, membranes were washed and incubated again with respective secondary antibodies, either goat anti-rabbit IgG at 1:2500 dilution (#7074S, Cell Signaling Technology) or goat anti-mouse IgG at 1:5000 dilution (#7076P2, Cell Signaling Technology) at room temperature for 1 h. Chemiluminescence (LumiGLO, Gaithersburg, MD, USA) and autoradiography were used to visualize the final reaction. Band densitometry was performed using ImageJ Software (NIH, Bethesda, MD, USA, http://imagej.nih.gov/ij/).

### Immunofluorescence, confocal microscopy, and image analysis

Immunofluorescence staining and confocal imaging on primary HBMECs wre performed according to our recently published protocol [19]. Briefly, after treatment with either human plasma Fgn (4 mg/mL) or vehicle control for 24 h, primary HBMECs were fixed with 4% paraformaldehyde (PFA) for 15 min at room temperature, washed with PBS, and permeabilized with PBST (PBS with 0.1% Triton X-100) for 20 min. Cells were blocked for non-specific binding using PBS containing 5% donkey serum (Sigma-Aldrich Cat. No.: D9663) for 1 h at room temperature, followed by overnight incubation at 4 °C with a rabbit anti-PECAM-1 (CD31) antibody (BiCell; # 01004) at 1:300 dilution and a rat anti-ZO-1 (Tjp1 C-terminus) antibody (BiCell, # 00236) at 1:300 dilution. Primary antibodies were diluted in PBS with 5% donkey serum. The next day, cells were rinsed quickly 3 × with PBS and then washed with PBS for 2 h at room temperature on a rocker. Cells were then incubated with donkey anti-rabbit (Invitrogen; # A-21206) and donkey anti-rat (Invitrogen; # A-48272) secondary antibodies, both at a 1:500 dilution for 2 h at room temperature on a rocker and protected from the light. Finally, cells were quickly rinsed with PBS 3 × to remove excess unbound secondary antibody, washed with PBS for 2 h at room temperature on a rocker, and then mounted with ProLong Glass antifade mountant with NucBlue stain (Invitrogen; # P36981).

Fluorescence images were collected using a 40 × water immersion objective on an Andor Dragonfly 202 (+ Leica DMI8 stand) high-speed confocal imaging platform equipped with solid-state 405, 488, 561, and 637 nm smart diode lasers. Images were acquired using a Zyla PLUS 4.2 Megapixel sCMOS camera. Confocal maximum projections were then analyzed to assess the levels of fluorescence specifically at the intercellular junctions, i.e., along the perimeter/contour of cells, where PECAM-1 and ZO-1 are located. Using ImageJ Fiji, the contour of various cells was individually hand-drawn using the segmented line tool. Then, by means of the straighten tool, the area along a 30-pixel wide contour was straightened and the normalized fluorescence intensity was determined by measuring the integrated density and normalizing it by the area spanned by each cell contour. Five different regions of interest, or fields of view, were analyzed per culture dish; and contours for 5 different cells were analyzed per field of view. Thereby, each individual datapoint represents the average values of all these measurements. Data is presented as the mean ± SEM of the average normalized integrated fluorescence density for each group. Statistically significant differences were evaluated using a parametric *t*-test with a significance set at *p < 0.05.

### Stable knockdown of DRP1 in primary HBMECs

We developed stable DRP1 knockdown cells by transducing shRNA(h) lentiviral particles (Santa Cruz, sc-43732-V) according to the manufacturer’s instruction. DRP1 shRNA (h) lentivirus particles containing 19–25 nucleotides (plus hairpin) shRNA were designed to knockdown the specific expression of DRP1. Briefly, cells were cultured in a 12-well plate (20,000 cells/well). The next day, the medium was replaced with polybrene (sc-134220) containing (5 µg/mL) fresh culture medium, and cells were transduced with an infection multiplicity of three (MOI = 3) overnight. The next day, the infection-containing culture medium was removed, and cells were washed with PBS, and then cultured with a complete medium (without Polybrene). After 24 h, the cells were split 1:3 and cultured for 72 h in complete medium, collected on a cell palette, lysed, and the cell lysates were prepared for western blot analysis.

### Statistical analysis

For quantitative proteomic data, we used a *t*-test analysis by grouping biological replicates and presenting pair-wise comparisons for fold-change: young, middle-aged, and old mice. The normalized abundance quantity of a biological replicate was calculated from an average of three experimental replicates. The data was shown as mean ± standard deviation (SD). Primarily, the data sets were calculated by the Shapiro–Wilk/D’Agostino-Pearson/Kolmogorov–Smirnov tests for normality followed by unpaired *t*-test with Welch correction for normally distributed data. When the data did not pass the normality test, a non-parametric Mann–Whitney test was employed as indicated in the figure legends. GraphPad Prism version 9.5.1 for Windows was used for statistical analysis, and *p* < 0.05 was considered statistically significant.

## Results

### DEPs involved in biological functions and CNS-related disease/disorder in mice cortical MVs with aging

**Fig. 1:**
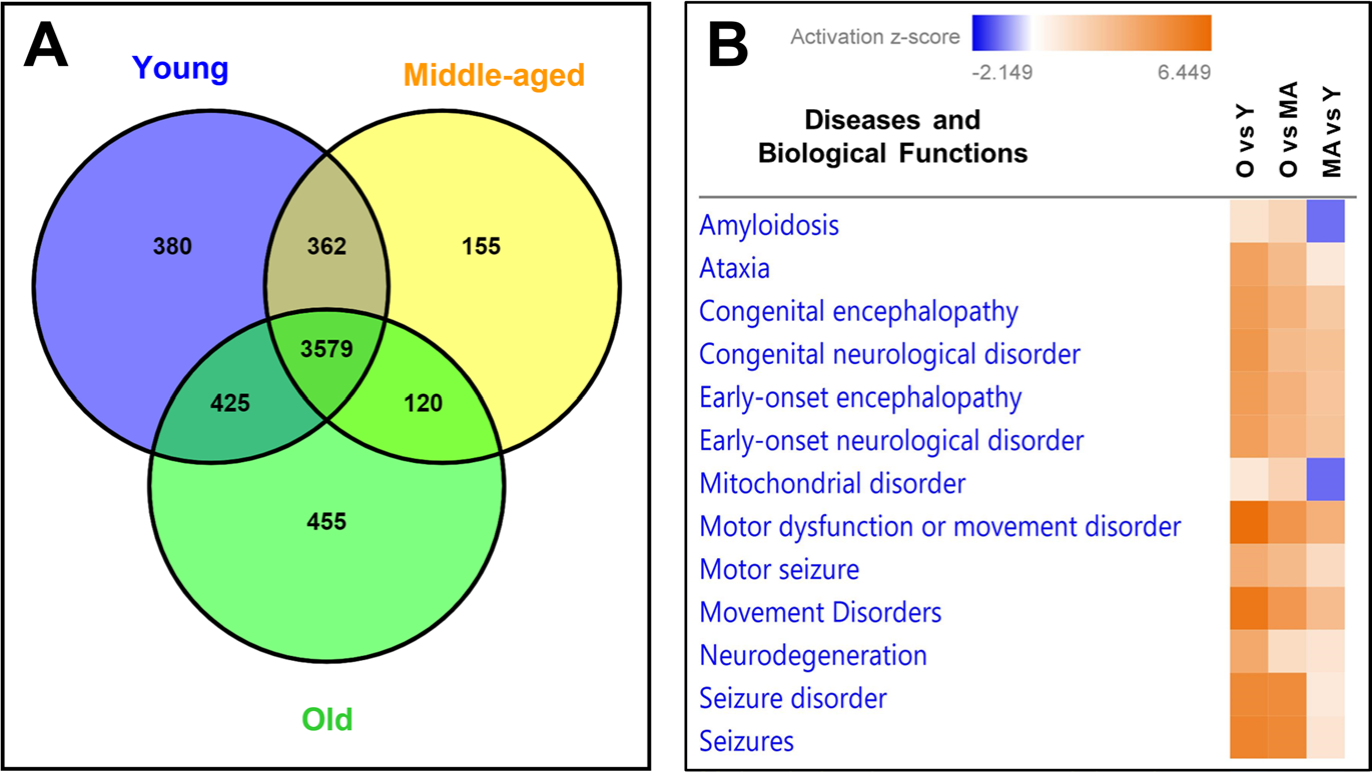
Differentially expressed proteins in mice cortical MVs and their involvement in diseases and biological functions with aging. **A** Venn diagram was drawn by using VENNY 2.1 (https://bioinfogp.cnb.csic.es/tools/venny/index.html) online web tool. Protein clustering in cortical MVs of young, middle-aged, and old mice by proteomic analysis. Relative protein abundance quantified by TMT-based proteomic study. **B** A comparison analysis depicting the diseases and biological functions associated with neuropathogenesis in cortical MVs of young, middle-aged, and old mice. The intensity of the heat map color corresponds to the activation *z*-score due to the involvement of upregulated and downregulated proteins. Age-matched, three males and three females were included in each group (*n* = 6/group) in this study. Y young, MA middle-aged, O old

We reported previously that 4746, 4216 and 4579 DEPs were quantified by proteomics in brain cortical MVs of young, middle-aged, and old mice, respectively [13]. Venn diagram indicated that 3579 proteins were common in all age groups; however, 380 (8%), 155 (3.7%), and 455 (10%) unique proteins were quantified in young, middle-aged, and old mice, respectively **(**Fig. 1A**)**. IPA analyses indicated that DEPs were involved in several CNS-related disorders and the dysregulation of biological functions in mice cortical MVs with aging (Fig. 1B).

### DEPs were involved in dysregulation of BBB and mitochondrial fission/fusion in mice cortical MVs with aging

**Fig. 2:**
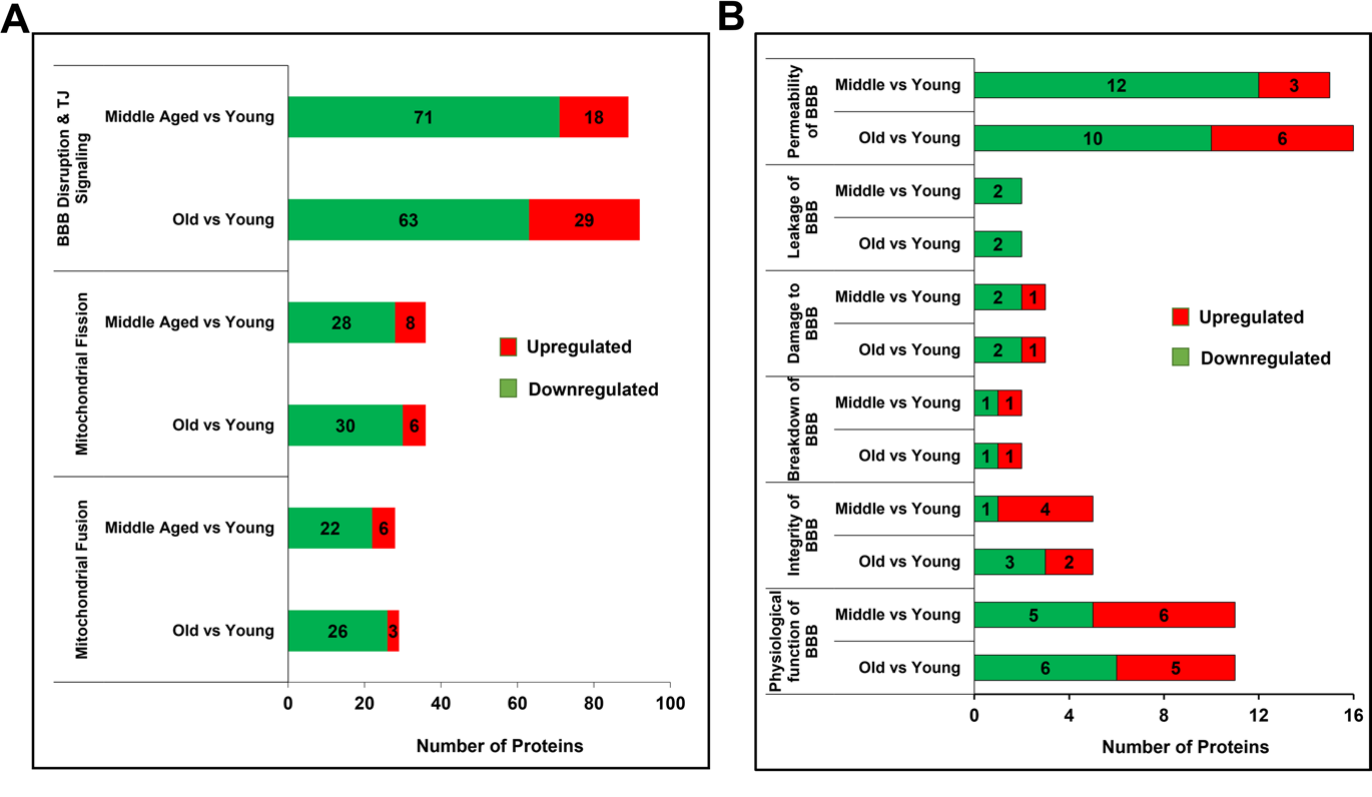
Differentially expressed proteins (DEPs) were involved in the dysregulation of BBB and mitochondrial fission/fusion in mice cortical MVs with aging. **A** IPA indicated the upregulated (red) and downregulated (green) proteins involved in BBB disruption and TJ signaling, mitochondrial fission/fusion in middle-aged and old vs. young mice’s cortical MVs. The DEPs and their fold change are listed in Supplementary Table 1. **B** More detailed bioinformatic analysis shows the number of proteins involved in the pathophysiology of BBB. Protein names and their fold change are listed in Supplementary Table 2. Age-matched, three males and three females were included in each group (*n* = 6/group) in this study

IPA indicated that the DEPs involved in BBB disruption and TJ signaling were mostly downregulated in middle-aged vs. young (~ 80%, 71/89) and old vs. young (~ 68%, 63/92) mice cortical MVs (Fig. 2A). The DEPs and their fold change are listed in Supplementary Table 1. Most of the downregulated proteins were common for both the middle-aged and old vs. young mice brain MVs. Among the upregulated DEPs, ACTC1, ACTB, F11R, PPM1L, SPTAN1, and VAMP3 increased in both middle-aged and old vs. young mice brain MVs. However, several cleavage stimulation factor subunits (CSTF1-3), cleavage- and polyadenylation-specific factors (CPSF1-4), AKT1, GPAA1, JAM2, MYH1, MHY11, MYL1, MYLK, PRKAR1A, TGFBR2, and VAPA were only upregulated in old vs. young mice brain MVs. Some proteins (CLDN11, JAM3, MYH14, NECTIN1, NAPB, PTEN, PPP2CA, PPP2R-2A, and -5B) were upregulated in middle-aged vs. young mice brain MVs (Supplementary Table 1)**.** More comprehensive IPA analysis indicated that proteins involved in permeability, leakage, damage, breakdown, integrity, and physiological function of BBB were differentially expressed in middle-aged and old mice cortical MVs (Fig. 2B). The DEPs and their fold change are listed in Supplementary Table 2. Mitochondrial fission–related proteins were mostly downregulated both in middle-aged vs. young (78%, 28/36) and old vs. young (83%, 30/36) mice cortical MVs. Similarly, DEPs involved in mitochondrial fusion were also mostly downregulated both in middle-aged vs. young (78%, 22/28) and old vs. young (90%, 26/29) mice cortical MVs (Fig. 2A). The DEPs and their fold change are listed in Supplementary Table 1.

### The abundance of Fgn was increased, but BBB- and mitochondrial fission/fusion–related proteins were decreased in cortical MVs of older than young mice

**Fig. 3:**
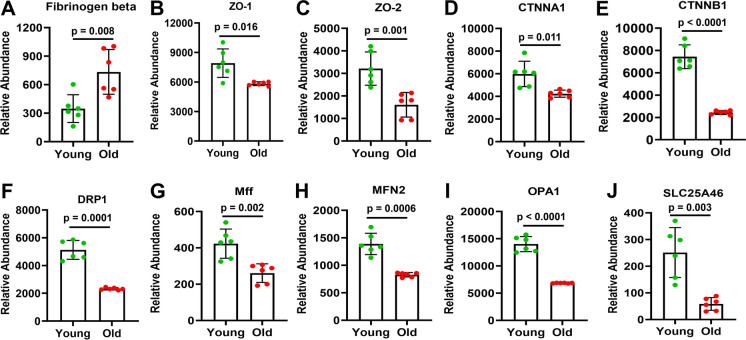
The abundance of fibrinogen beta, BBB- and mitochondrial fission/fusion–related proteins in the cortical MVs of old vs. young mice. Altered age-specific expression of fibrinogen beta (panel **A**), BBB-related (panels **B**–**E**), and mitochondrial fission/fusion–related (panels **F**–**J**) proteins in mouse’s cortical MVs. The abundant expression of different proteins that exhibited differences between old vs. young mice is shown in bar graphs. Graphs show mean ± SD of relative abundance, with significant differences between groups presented as indicated. Proteins presented in different panels passed the Shapiro–Wilk/D’Agostino-Pearson/Kolmogorov–Smirnov normality tests followed by an unpaired *t*-test with Welch correction. Age-matched, three males and three females were included in each group (*n* = 6/group)

Proteomic data indicated that the expression of fibrinogen beta was significantly higher in cortical MVs of older than young mice (Fig. 3A). On the other hand, BBB-related ZO-1, ZO-2, CTNNA1, and CTNNB1 proteins were significantly less expressed in cortical MVs of older than young mice (Fig. 3B–E). Similarly, several mitochondrial fission/fusion–related proteins such as DRP1, Mff, MFN2, OPA1, and SLC25A46 were significantly less expressed in cortical MVs of older than young mice (Fig. 3F–J).

### Protein-network analysis showed that high expression of Fgn was linked with downregulated expression of BBB and TJ-signaling proteins in the cortical MVs of old vs. young mice

**Fig. 4:**
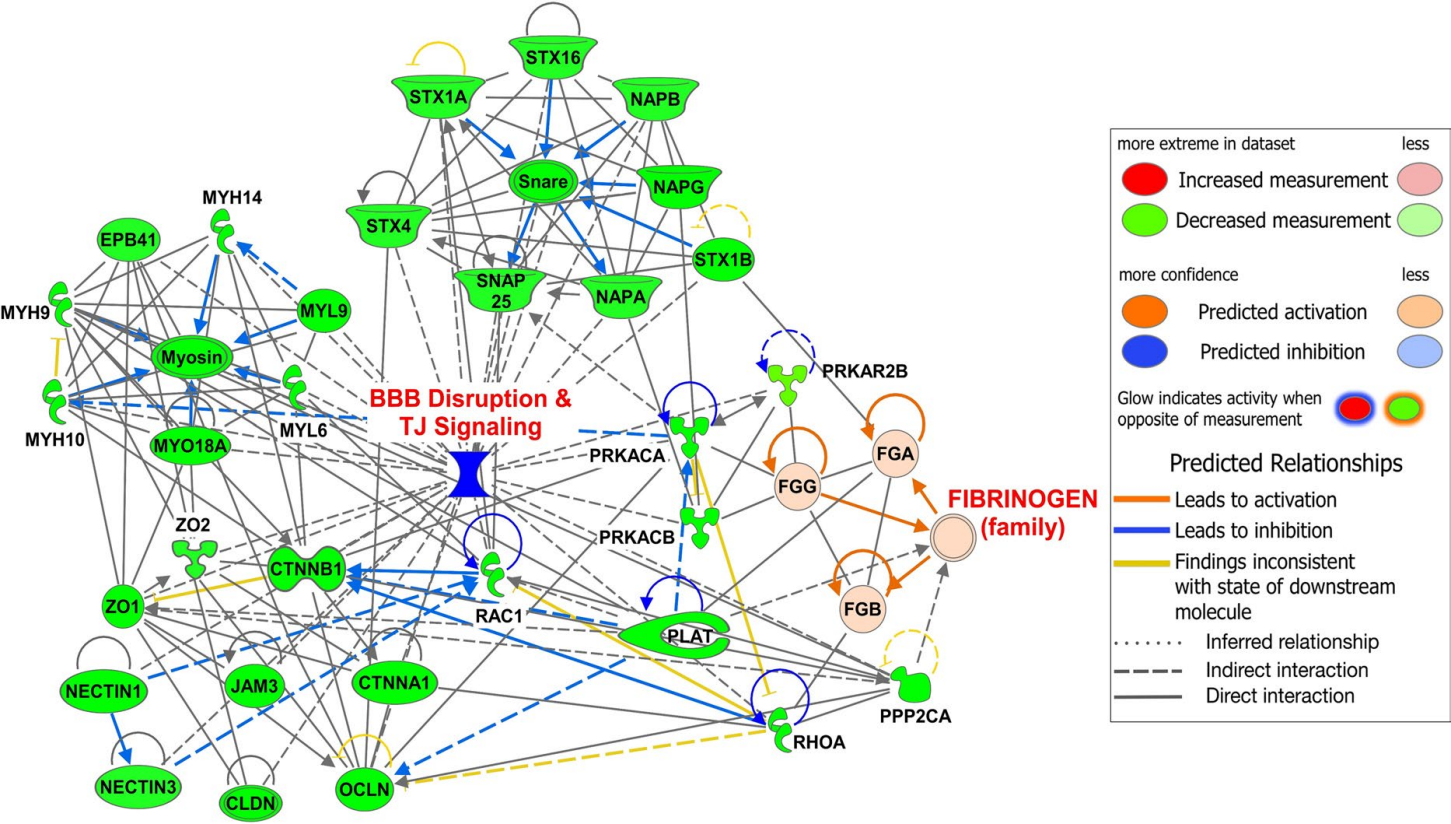
Protein network analysis of Fgn and BBB disruption and TJ-signaling proteins in the cortical MVs of old vs. young mice. Graphical presentation of the interaction network between upregulated Fgn (red) and downregulated (green) BBB disruption and TJ-signaling pathways–related proteins in cortical MVs of old vs. young mice were developed by IPA. STX1B, PRKAR2B, PRKACA, PRKACB, PLAT, RHOA, and PPP2CA proteins are directly linked to Fgn. FGA, FGB, and FGG are three different isoforms of Fgn. The protein symbols used in this network analysis and their fold change are listed in Supplementary Table 3. Age-matched, three males and three females were included in each group (*n* = 6/group) in this study

Functional interactions among the Fgn family proteins (FGA, FGB, and FGG) and several BBB and TJ-signaling proteins were analyzed by IPA. Interestingly, decreased expression (indicated by green) of syntaxin-1B (STX1B), cAMP-dependent protein kinase type II-beta regulatory subunit (PRKAR2B), cAMP-dependent protein kinase catalytic subunit-alpha (PRKACA), -beta (PRKACB), tissue-type plasminogen activator (PLAT), serine/threonine-protein phosphatase 2A catalytic subunit alpha isoform (PPP2CA), and Ras homolog family member A (RHOA) proteins interacted directly (indicated by solid black lines) with Fgn family proteins. Simultaneously, these proteins were directly linked with several TJ (ZO-1, ZO-2, JAM3, OCLN, CLDN), TJ-contractile (Myosin, MYO18A, MYL6, MYL9, MYH9, MYH10, and MYH14), and adherens junction (CTNNA1, CTNNB1, NECTIN1, NECTIN3) proteins, which were downregulated in old vs. young mice cortical MVs. Interactome analysis also indicated that TJ-signaling proteins, RHOA and RAC1, were also downregulated in old vs. young mice MVs. Solid or broken blue arrows indicate the inhibition influence of one protein by others (Fig. 4). The proteins used in this network analysis and their fold changes are listed in Supplementary Table 3.

### Interactome analysis showed that high expression of Fgn was associated with reduced expression of mitochondrial fission/fusion–related proteins in the cortical MVs of old vs. young mice

**Fig. 5:**
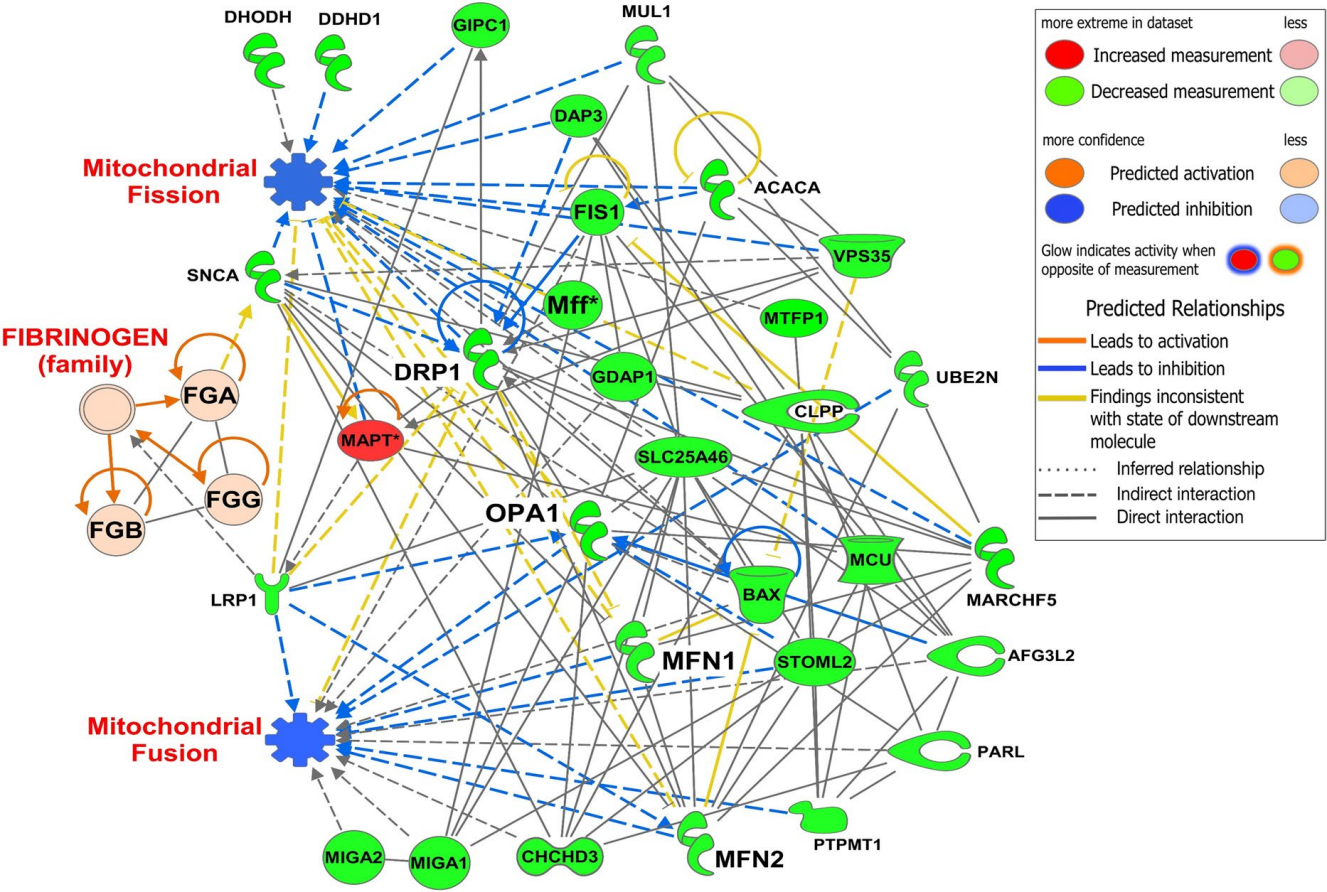
Protein network analysis of Fgn and mitochondrial fission/fusion–related proteins in cortical MVs of old vs. young mice. Protein–protein interaction between downregulated (green) mitochondrial fission/fusion-related proteins and upregulated Fgn (red) in cortical MVs of old vs. young mice was generated by IPA. Proteins SNCA and LRP1 are directly linked to Fgn. Concurrently, several mitochondrial fission/fusion–related proteins are directly or indirectly associated with SNCA and LRP1, indicated by continuous and broken lines with different colors. The protein symbols used in this network analysis and their fold change are listed in Supplementary Table 4. Age-matched, three males and three females were included in each group (*n* = 6/group) in this study

Protein–protein interaction by IPA showed that a high abundance of FGA, FGB, and FGG (indicated by red) was linked with several downregulated mitochondrial fission/fusion–related proteins (indicated by green) in cortical MVs of old vs. young mice. The network analysis revealed that low density lipoprotein receptor-related protein 1 (LRP1) and synuclein alpha (SNCA) were linked with Fgn family proteins. Both LRP1 and SNCA were directly or indirectly associated with the downregulation of mitochondrial fission/fusion proteins. Protein network analysis indicated that LRP1 leads to the inhibition of mitochondrial fusion proteins, OPA1, and MFN2 (broken blue line). Similarly, in this network, SNCA is linked with DRP1, OPA1, MFN1/2, and other mitochondrial fission/fusion proteins (indicated by broken blue and black lines). Interestingly, the high expression of microtubule-associated protein tau (MAPT; indicated by red) is directly linked with DRP1, LRP1, SNCA, and several mitochondrial fission/fusion proteins in cortical MVs of old vs. young mice (Fig. 5). The proteins used in this interactome analysis and their fold changes are listed in Supplementary Table 4.

### Increased concentration of human plasma–derived Fgn changed cell morphology and induced cytotoxicity in primary HBMECs

**Fig. 6:**
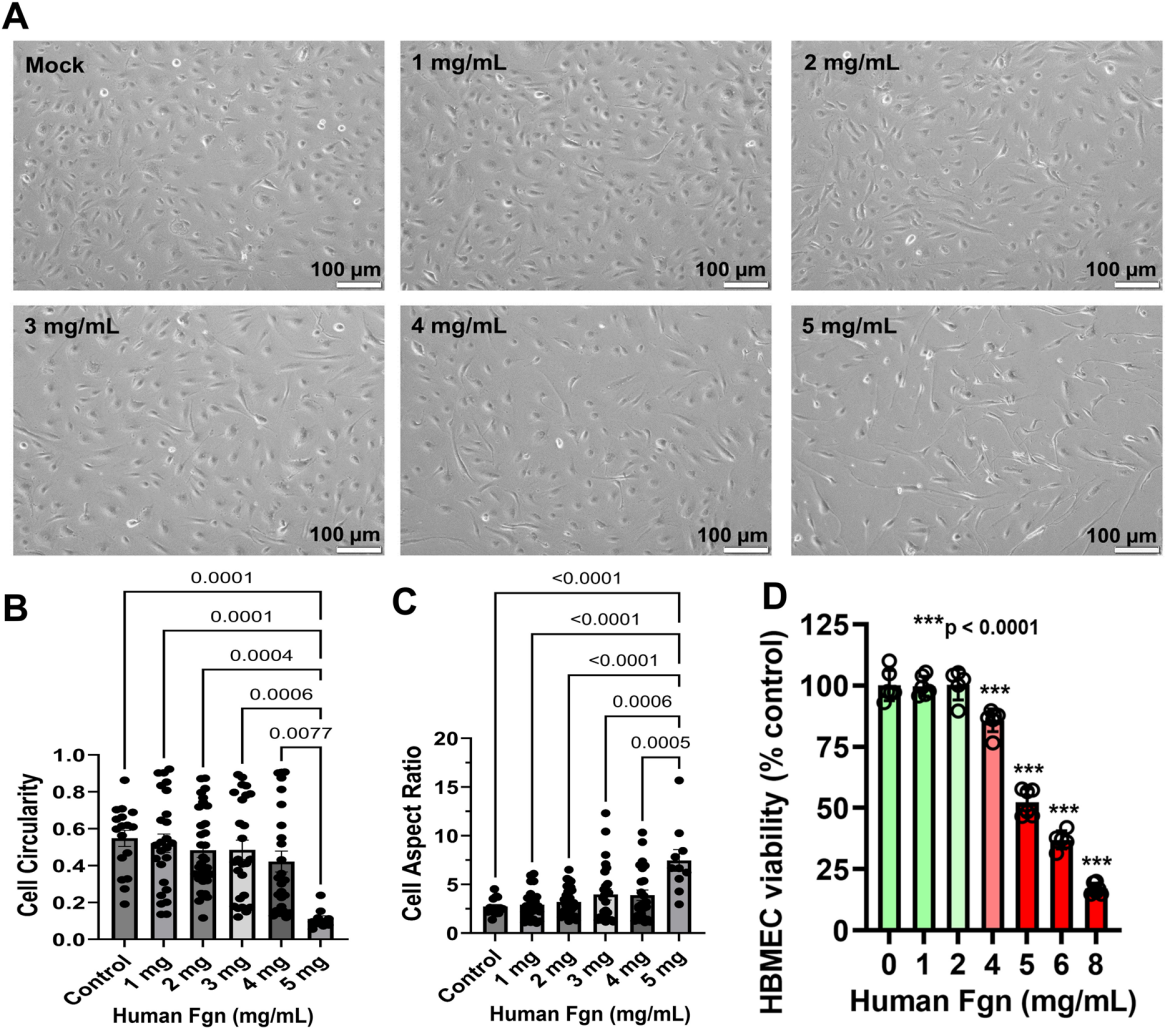
High expression of fibrinogen changes the morphology and induces cytotoxicity in primary HBMECs. **A** Equal numbers (5 × 10^4^) of primary HBMECs were cultured in a 6-well plate. After 48 h, cells were cultured with fresh medium and treated with indicated concentrations of human plasma Fgn for 24 h. Pictures were taken the next day with bright field under microscopy, and representative pictures of the treated or untreated (Mock) cells were compared for change in morphology. **B**–**C** We measured the cell circularity and the cell aspect ratio using ImageJ Fiji software. Images were transferred from RGB to 16-bit grayscale and then subjected to automated contrast enhancement. Nearly 10–20 cells were manually segmented using the freehand selection tool in each field of view. Using the analyze particles tool, objects were automatically detected, and their morphology was characterized by measuring various shape descriptors, including aspect ratio and circularity. Aspect ratio refers to the ratio of the major to minor axis from an elliptical fit to the cell shape; while circularity is a parameter that ranges from 0 to 1, with 1 being a perfect circle. **D** To measure the cell viability, equal numbers (5 × 10^3^) of primary HBMECs were cultured in a 96-well plate. The next day, cells were treated with indicated concentrations of human plasma Fgn for 24 h, and cell viability was measured by MTT-based assay. Significant change of cell viability with increasing concentration of Fgn is indicated by asterisks. The experiment was repeated three times (*n* = 3), and in each time of the experiment, five experimental replicates were performed. The data sets were passed both the Shapiro–Wilk and Kolmogorov–Smirnov normality tests and followed by an ordinary one-way ANOVA test for multiple comparisons of normally distributed data

We observed that HBMECs morphology changed from normal architecture to more elongated forms with increasing concentrations of Fgn. Cell proliferation rate decreased with increasing Fgn concentration, and most cells were spindle-shaped upon exposure to 4 mg/mL or higher concentration of Fgn **(**Fig. 6A). We measured the circularity of cells and the cell aspect ratio using ImageJ software. The results clearly indicated that cell circularity significantly decreased with increasing concentration of Fgn **(**Fig. 6B**)**. On the other hand, the cell aspect ratio significantly increased with increasing concentration of Fgn (Fig. 6C**)**. Also, cell viability was significantly decreased when treated with 4 mg/mL or higher concentration of Fgn (Fig. 6D).

### Human plasma–derived Fgn increased BBB permeability possibly due to the downregulation of BBB-related TJ proteins in primary HBMECs

**Fig. 7:**
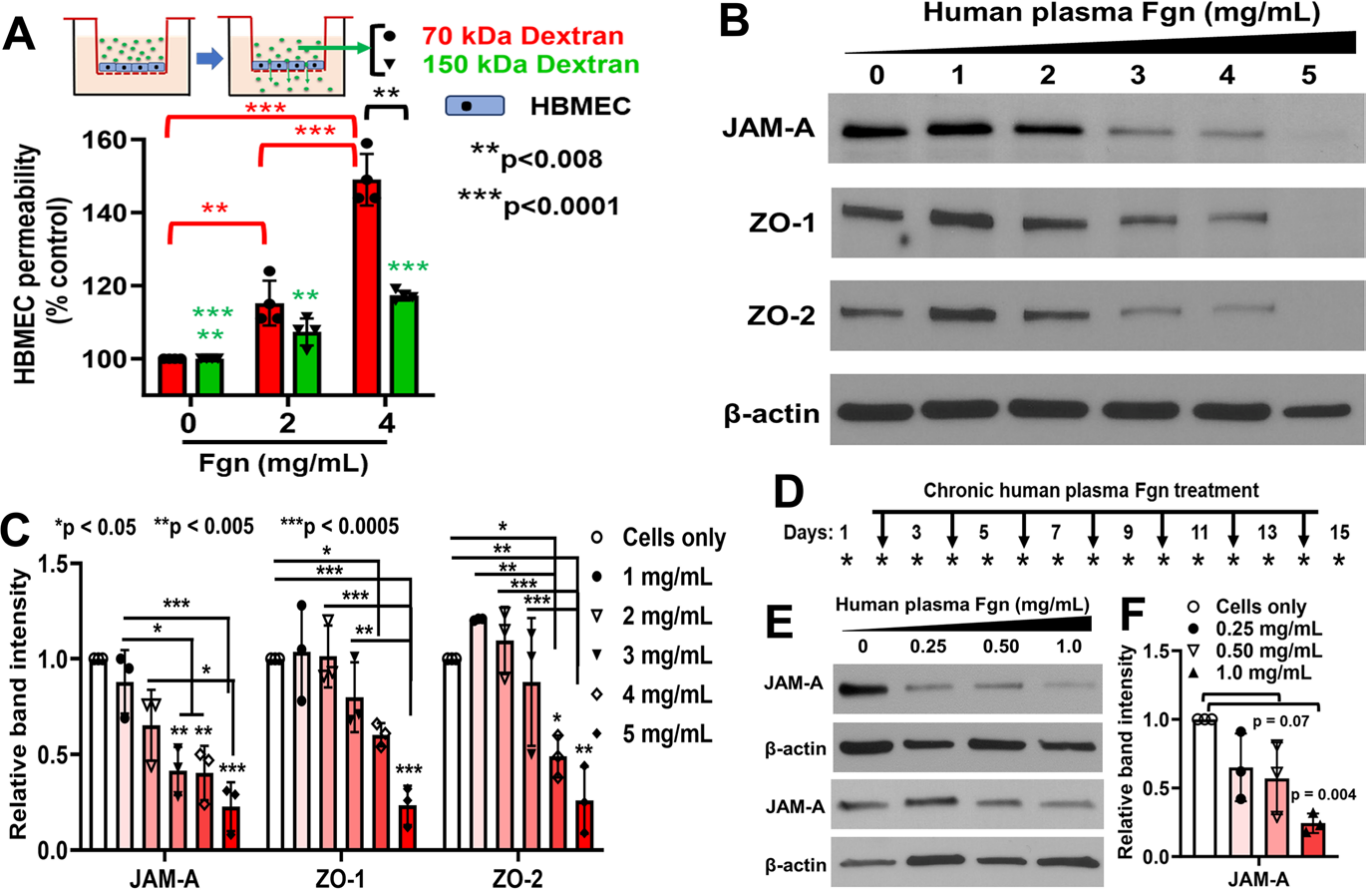
Human plasma Fgn downregulated the expression of BBB-related proteins in primary HBMECs. **A** In vitro BBB permeability was measured by transwell migration assay. Primary HBMECs (2 × 10^5^) were cultured in the upper chamber of the transwell and allowed to grow for 72 h to form a cell monolayer with a complete BBB. The BBB permeability was measured after 24 h exposure to Fgn. A schematic diagram of the transwell-based BBB permeability assay is presented at the top. Graph showing the BBB permeability efficiency of fluorescent-labeled 70 kDa and 150 kDa molecular weight of FITC-Dextran. In each time of the experiment, four experimental replicates were performed. The data sets have passed the Shapiro–Wilk normality test followed by an ordinary one-way ANOVA test for multiple comparisons of normally distributed data. **B**–**C** Equal numbers (5 × 10^4^) of primary HBMECs were cultured in a 6-well plate. The next day, cells were treated with the indicated concentrations of human Fgn for 24 h, and BBB-related proteins, JAM-A, ZO-1, and ZO-2 were detected by western blots. The relative band intensities were quantified by ImageJ software and compared from three independent experiments (*n* = 3). The data sets have passed the Shapiro–Wilk normality test followed by an ordinary one-way ANOVA test for multiple comparisons of normally distributed data. **D** Protocol for the repeated treatments of HBMECs with low concentrations of human plasma Fgn. Cells were treated every 2 days (indicated by black arrows) up to 15 days with 0.25, 0.5, and 1.0 mg/mL of Fgn. **E**–**F** At the end of the experiment, cells were lysed and the expression of JAM-A was measured by western blots. The relative band intensities were quantified by the ImageJ software and compared from three independent experiments (*n* = 3). The data passed the Shapiro–Wilk normality test followed by an ordinary one-way ANOVA test for multiple comparisons of normally distributed data

**Fig. 8:**
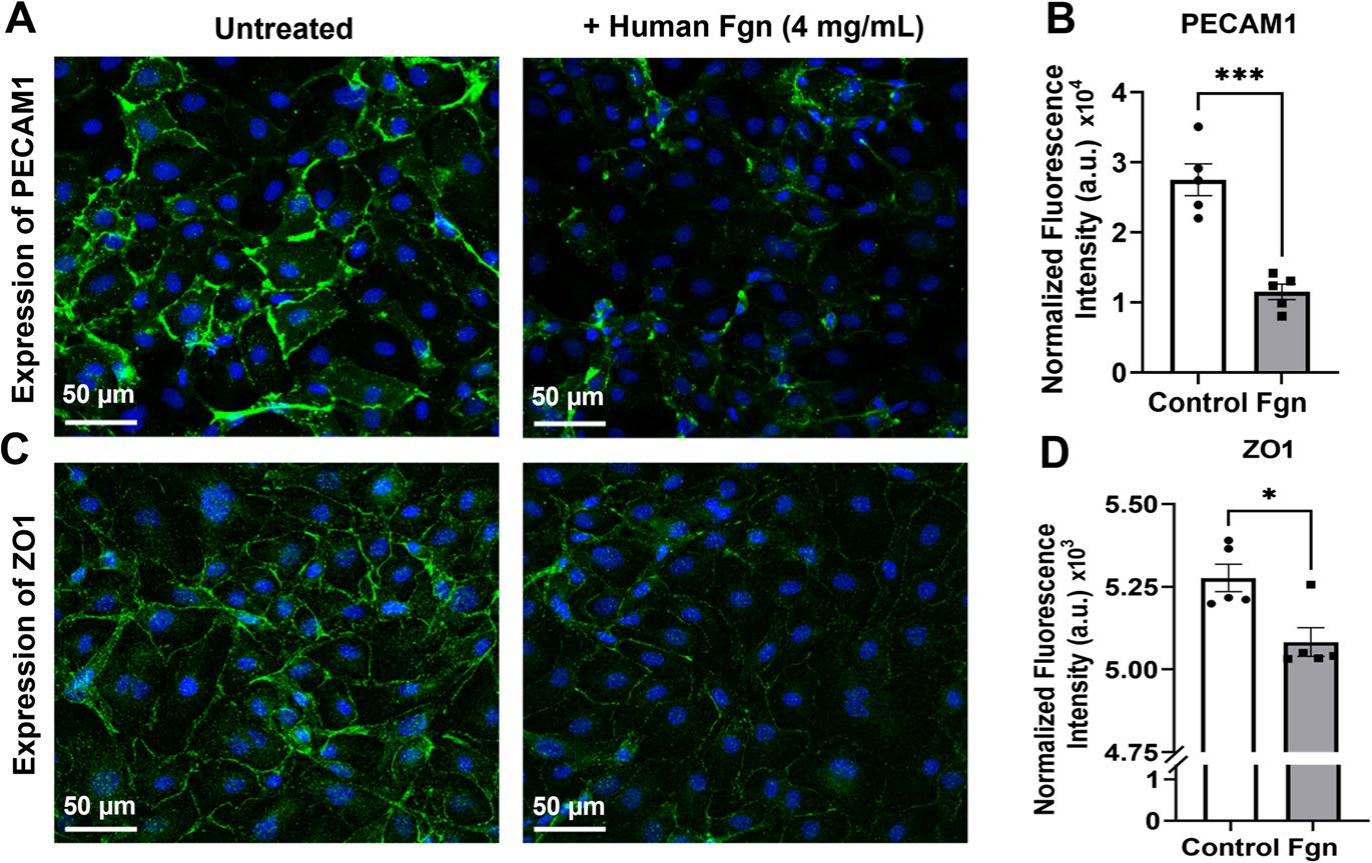
Immunofluorescence studies indicated the downregulation of BBB-related proteins by human plasma Fgn in primary HBMECs. Primary HBMECs (1 × 10.^6^) were cultured in 35-mm glass-bottom dishes for 24 h. The next day, cells were treated with human plasma Fgn for 24 h. After treatment, cells were fixed, and the expressions of the indicated proteins were detected by immunofluorescence technique under confocal microscopy. **A** and **B** The expression of PECAM1 was compared in Fgn-treated and untreated cells, and the fluorescence intensity only in the cell membrane regions was quantified from randomly selected cells (*n* = 5) in each group and compared. Similarly, **C** and **D** the expression of ZO1 was compared in Fgn-treated and untreated cells, and the fluorescence intensity was quantified from randomly selected cells only in the cell membrane regions (*n* = 5 in each group) and compared. The data set passed both the Shapiro–Wilk and D’Agostino-Pearson omnibus normality tests for PECAM-1 and the D’Agostino-Pearson normality test for ZO-1. An unpaired *t*-test with Welch correction was performed for normally distributed data. *p < 0.012, ***p < 0.0002

Transwell migration-based BBB permeability assay indicated that even 2 mg/mL of Fgn significantly increased BBB leakage. BBB permeability further increased with increasing concentration (4 mg/mL) of Fgn. Interestingly, due to the smaller size of FITC-dextran, we observed that the percent increase of BBB permeability by 70 kDa FITC-dextran was much higher than the percent increase by 150 kDa FITC-dextran. A significant increase of barrier permeability even with 150 kDa FITC-dextran indicates that moderate to high concentration of Fgn could cause severe leakage of both paracellular (BBB), or transcellular pathways (Fig. 7A). Western blot analysis indicated the dose-dependent decrease in TJ proteins such as JAM-A, ZO-1, and ZO-2 with increasing concentration of Fgn (Fig. 7B–C). JAM-A expression was significantly reduced when cells were treated with 3 mg/mL or higher concentrations of Fgn. However, for ZO-1 and ZO-2, the expression was significantly reduced at 4 mg/mL or higher concentrations of Fgn (Fig. 7C). Interestingly, the expression of occludin and claudin-5 was significantly increased when cells were treated with 2 mg/mL of Fgn; however, the expression of VE-cadherin was significantly decreased both at 2 mg/mL and 4 mg/mL of Fgn **(**Supplementary Fig. 1A–B). We studied the long-term effect of low concentrations of Fgn on HBMECs. Cells were treated every 2 days up to 15 days with 0.25, 0.5, and 1.0 mg/mL of Fgn (Fig. 7D). Interestingly, the expression of tight junction protein, JAM-A was decreased due to chronic treatment of low concentrations of Fgn on HBMECs (Fig. 7E–F). Immunofluorescence further supported our western blot results. Platelet endothelial cell adhesion molecule 1 (PECAM1; also known as CD31), which regulates endothelial junctional integrity, was significantly decreased in Fgn-treated HBMECs (Fig. 8A–B). Moreover, we observed discontinued and significantly less membrane staining of ZO-1 in Fgn-treated cells compared with untreated cells (Fig. 8C–D).

### Human plasma–derived Fgn downregulated the expression of mitochondrial fission/fusion–related proteins in primary HBMECs

**Fig. 9:**
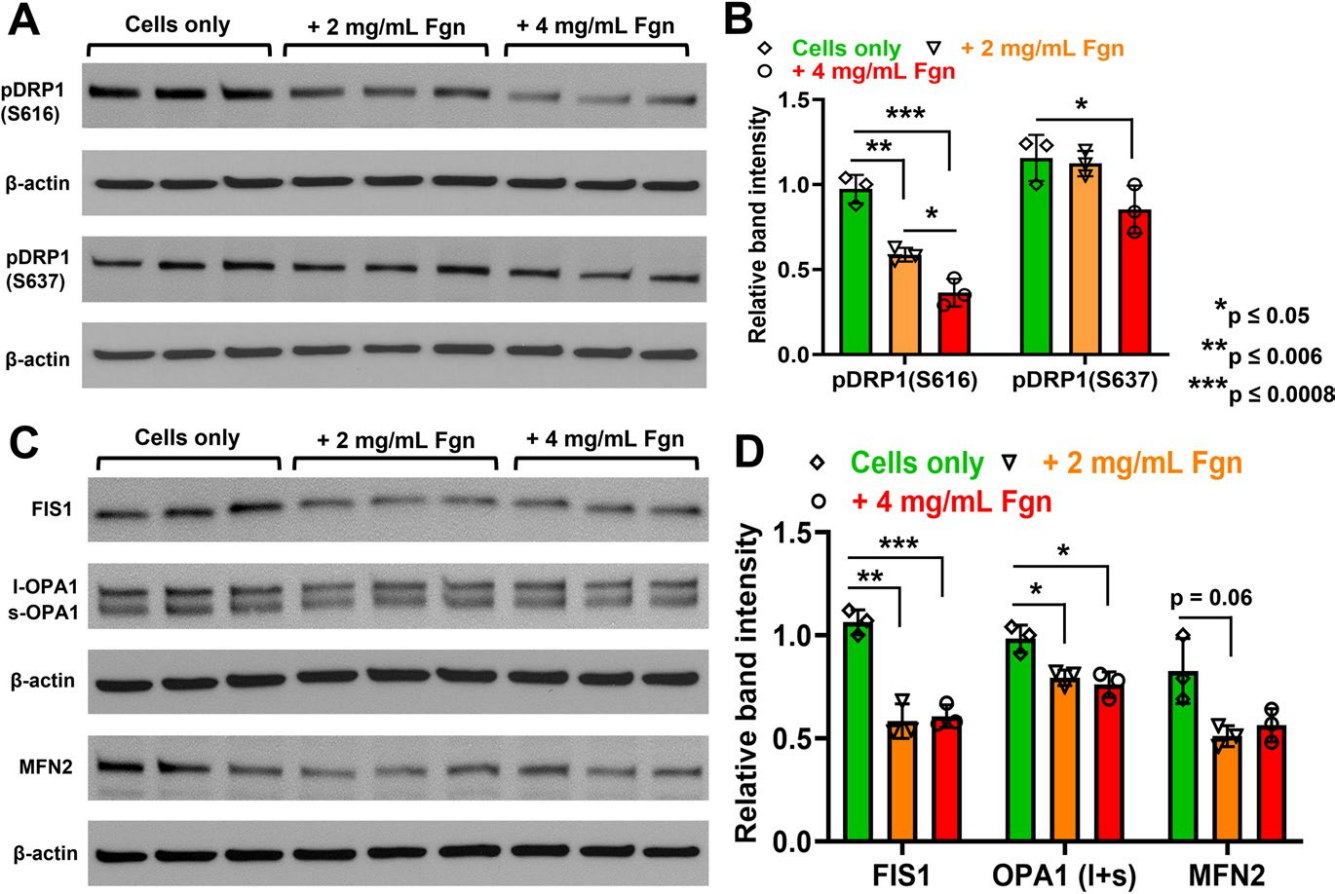
Human plasma Fgn downregulated the expression of mitochondrial fission/fusion–related proteins in primary HBMECs. Primary HBMECs (5 × 10.^4^) were cultured in a 6-well plate for 24 h. The next day, cells were treated with human plasma Fgn for 24 h. After treatment, cells were harvested and lysed, and expressions of indicated proteins were detected by western blots. **A**–**B** Expression of phosphorylated DRP1 (both S616 and S637) and β-actin as an internal control was compared in Fgn-treated and untreated cells and band intensity was quantified by ImageJ software and compared. Similarly, **C**–**D** expressions of FIS1, OPA1, MFN2, and β-actin were compared in Fgn-treated and untreated cells, and band intensity was quantified by the ImageJ software and compared. The relative band intensities were quantified by the ImageJ software and compared from three independent experiments (*n* = 3). The data set passed both the Shapiro–Wilk and Kolmogorov–Smirnov normality tests, and an unpaired *t*-test with Welch correction was performed for normally distributed data. l-OPA1 large subunit of OPA1, s-OPA1 small subunit of OPA1

We observed that mitochondrial fission protein pDRP1 (S616) was significantly decreased even at a low concentration of 2 mg/mL of Fgn and was further reduced when cells were treated with 4 mg/mL of Fgn. Interestingly, the expression of pDRP1 (S637) was significantly decreased using only 4 mg/mL of Fgn (Fig. 9A–B). Mitochondrial fission 1 protein (FIS1) was also significantly decreased even at a low concentration of 2 mg/mL of Fgn. Both large and small subunits of mitochondrial dynamin-like 120 kDa protein, known as OPA1, were decreased with increasing concentration of Fgn in HBMECs. However, another mitochondrial fusion protein, mitofusin-2 (MFN2), was not significantly decreased with increasing concentration of Fgn in HBMECs (Fig. 9C–D).

### Knockdown of DRP1 downregulated BBB- and mitochondrial fission/fusion–related protein expression in primary HBMECs

**Fig. 10:**
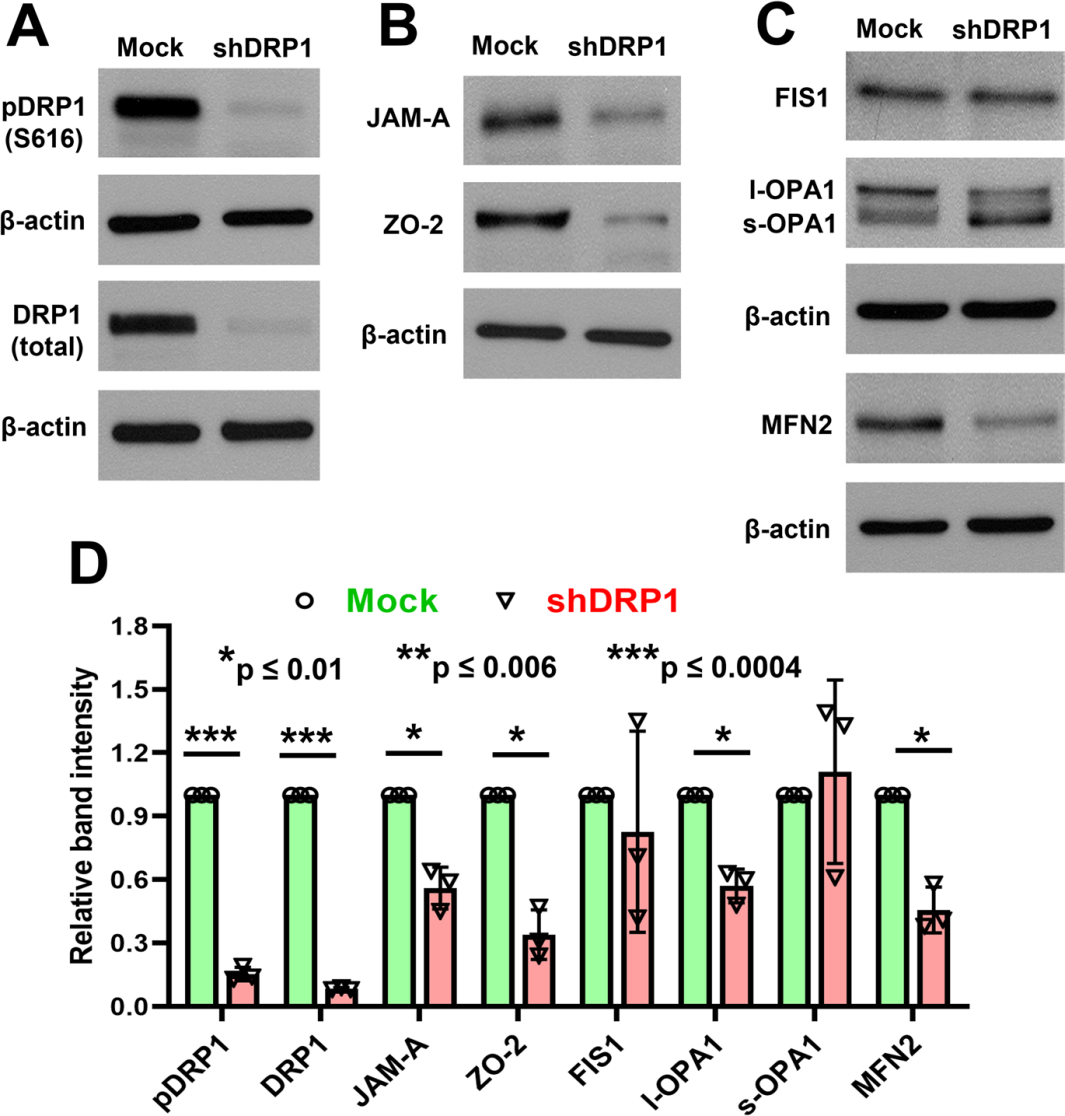
Knockdown of DRP1 downregulated the expression of BBB- and mitochondrial fission/fusion–related proteins in primary HBMECs. The DRP1 expression was knocked down by lentivirus expressing shRNAs against DRP1. **A** An expression of both pDRP1 and total DRP1 in DRP1 knock-down (shDRP1) and control cells (mock) is shown by western blots. Similarly, **B**–**C** a significant decrease of BBB (JAM-A and ZO-2) and mitochondrial fusion (l-OPA1 and MFN2) proteins in shDRP1 and mock cells are shown by western blots. **D **The relative band intensities were quantified by the ImageJ software and compared from three independent experiments (*n* = 3). The data set passed both the Shapiro–Wilk and Kolmogorov–Smirnov normality tests, and an unpaired *t*-test with Welch correction was performed for normally distributed data

After the stable knockdown of DRP1 by lentiviral expressing shRNA against DRP1 (shDRP1), we observed that nearly 80–90% of total DRP1 and pDRP1 (S616) were downregulated in shDRP1 cells (Fig. 10A and D). Concurrently, BBB-related TJ-proteins (JAM-A and ZO-2) were significantly decreased in shDRP1 cells compared with the control (mock) (Fig. 10B and D). The expression of FIS1 and the small subunit of OPA1 (s-OPA1) were not significantly changed; however, the large subunit of OPA1 (l-OPA1) and MFN2 was significantly decreased in shDRP1 cells compared to the Mock (Fig. 10C and D).

## Discussion

**Fig. 11:**
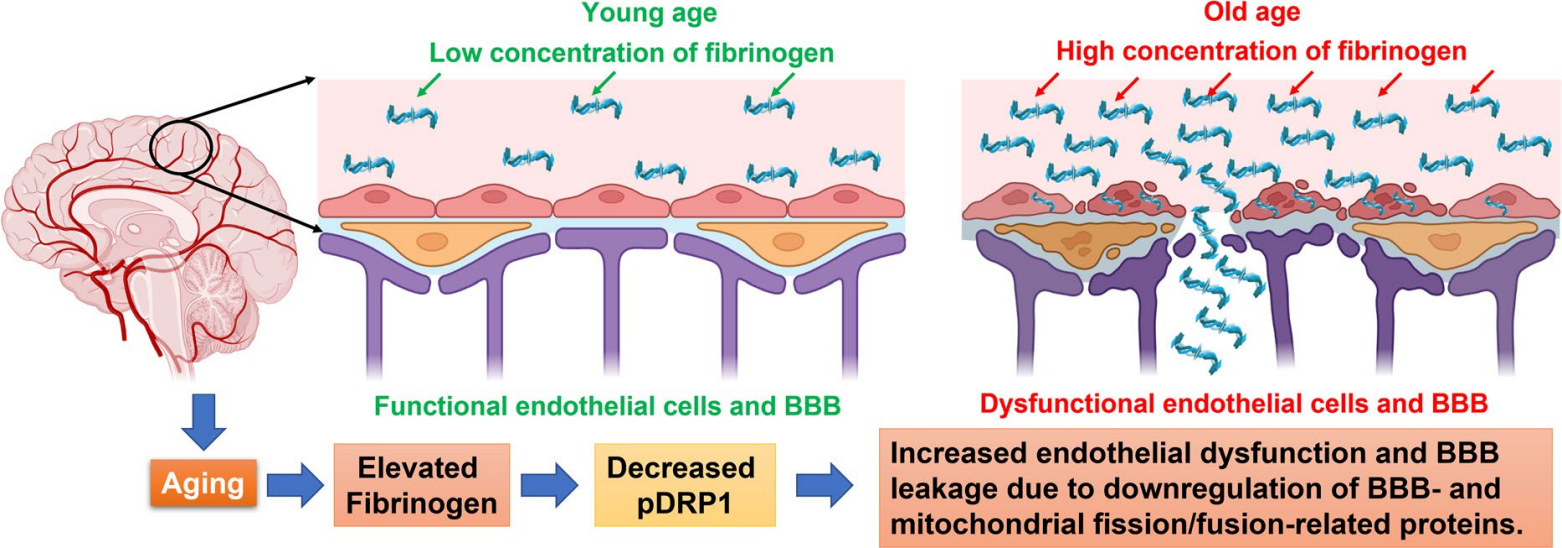
High expression of Fgn in mice cortical MVs with aging-induced mitochondrial-dependent endothelial dysfunction and BBB leakage via a DRP1-dependent pathway. Putative events leading to endothelial/BBB dysfunction in the brain microvasculature with aging. This study indicated that the elevated expression of Fgn decreased mitochondrial fission protein, DRP1, which induced brain endothelial dysfunction and BBB dysregulation. The figure was generated with the help of BioRender.com

Brain aging is a major risk factor in the progression of cognitive diseases and vascular dementia. Cerebral small vessel disease, a specific cause of subcortical vascular dementia, is a major cause of cognitive impairment [21–24] and may account for approximately 50% of all dementias worldwide [21, 25, 26]. We investigated normal brain aging in cortical MVs in mice up to 21 months of age and found that DEPs in cortical MVs were involved in several CNS-related disorders (early-onset neurological disorder, motor dysfunction or movement disorder, and seizure disorder) and the dysregulation of biological functions (mitochondrial dysfunction, neurodegeneration, and amyloidosis). Also, we established a link between neuroinflammatory protein, Fgn, and BBB- and mitochondrial fission/fusion–related proteins in cortical MVs with aging. Protein–protein network analysis showed that high expression of Fgn was associated with downregulated expression of BBB- and mitochondrial fission/fusion–related proteins in cortical MVs with aging, which may contribute to the escalation of this microvasculature vulnerability to ongoing damage, dysfunction, and increased susceptibility to brain injury and disease. To explore the mechanism, we observed that elevated human plasma Fgn altered cell morphology, induced cytotoxicity, and BBB-permeability possibly due to downregulation of BBB- and mitochondrial fission/fusion–related proteins via a DRP1 dependent pathway in primary HBMECs (Fig. 11—schematic).

Disturbing observations of extensive BBB leakage in older patients were first reported in the 1970s [27], and BBB breakdown is now a promising biomarker in normal aging [11, 12]. The cerebrospinal fluid/serum ratio of albumin is significantly increased with aging, which is now a reliable surrogate marker of increased BBB leakage [28, 29]. However, how the BBB permeability influences brain function in aging is still unknown. Recent studies on rodents have reported that aging aggravated the brain microvascular damage and BBB disruption due to decreased expression of TJ proteins [30–34]. Recently, we reported that several antioxidant proteins and almost all the glycolytic enzymes are decreased in cortical MVs of old mice [13] and other groups showed that inhibition of the glycolytic enzyme, GAPDH, produced higher BBB permeability and barrier dysfunction [35]; the same effect was triggered by overproduction of ROS in endothelial mitochondria [36]. In our study, several hallmark TJ, TJ-contractile, TJ-signaling, and adherens junction proteins were downregulated in cortical MVs of both middle-aged (12–14 months) and old (20–21 months) mice. These surprising results in middle-aged mice indicate that BBB dysfunction appears earlier than has been anticipated, possibly indicating that it is among the earliest known markers of aging in the mouse brain. The onset of BBB dysfunction at around 12–14 months links with other early biological hallmarks of aging, including the typical onset of reproductive senescence in female mice, as well as the earliest appearance of mild cognitive impairment in various behavior tasks [37–41].

Interestingly, our IPA-based investigation indicated that some unique proteins were upregulated only in cortical MVs of old mice, and those proteins are possibly correlated with more BBB dysregulation in old than in middle-aged mice. For example, several cleavage-stimulating factor subunits: (CSTF)-1 to CSTF-3, and cleavage- and polyadenylation-specific factor (CPSF)-1 to CPSF-4, involved in the cleavage of newly synthesized mRNAs were upregulated only in cortical MVs of old mice. Moreover, AKT1, TGFβR2, JAM2, and myosin light chain kinase (MYLK) were also upregulated only in cortical MVs of old mice. It has been reported that AKT1 levels increase due to brain injury [42] and PTEN/AKT1 pathway controls BBB permeability [43]. BBB breakdown is linked with hyperactivation of TGFβ signaling in astrocytes in aging humans and rodents [44]. Moreover, elevated JAM2 promotes lymphocyte transendothelial migration [45] and increased MYLK activity causes endothelial hyperpermeability and endothelial barrier dysfunction [46].

Recently, BBB breakdown has been linked to mitochondrial dysfunction in endothelial cells in stroke, neurological diseases, and aging [25, 47, 48]. Aging-related decline in mitochondrial turnover caused by reduced mitochondrial fission and fusion seems to be a particularly crucial factor for neuropathogenesis [49–51]. In healthy cells, mitochondrial fusion delivers a coordinated internal means for translocating metabolites and intramitochondrial mixing during biogenesis, whereas mitochondrial fission assists the equal distribution of mitochondria into daughter cells and permits selective degradation of damaged mitochondria through mitophagy [52, 53]. It was shown that MFN2 and DRP1 genes are reduced in the skeletal muscle of aging individuals [54]. Nevertheless, it has become clear that these protective mechanisms are significantly impaired in the aging process [49, 50]. Our current findings show that almost all the key fission/fusion-related proteins were downregulated in cortical MVs with aging.

A mechanistic understanding of the biological consequences of BBB breakdown is critical to recognize whether the relationship between mitochondrial dysfunction, BBB leakage, and cognitive impairment is unrelated, correlative, or causal. The normal aging process in the healthy brain is linked to a decrease in physiological function possibly due to the constant increase in neuroinflammation [55]. Clinical studies have reported that Fgn is an abundant protein in human blood plasma at concentrations ranging from 1.5–4 mg/mL with a normal half-life of 3–5 days, and the plasma concentration of Fgn increased progressively with age in healthy subjects [56–60]. Although the neuroinflammatory protein, Fgn, has shown an age-related increase in blood, which has been documented in major epidemiological studies [56–60], little is known about the pathological mechanism(s) of this increase in Fgn levels with age. Fgn infiltration and BBB dysfunction are characteristic features of several neurological diseases [61–64] and Fgn has been detected in the frontal cortex and hippocampal regions of Alzheimer’s disease brain tissue [65]. Fgn is one of several pathogenic factors of the cerebral endothelium that might impact BMEC function and BBB permeability [66, 67]. Several integrins (α_5_β_1,_ α_v_β3, and α_v_β5) [68–71] and intercellular adhesion molecule-1 (ICAM-1) [72, 73] are known Fgn receptors expressed on the endothelial cells, and an increase in ICAM-1 was attributed to stimulation by inflammatory cytokines and oxidant species in HBMECs [74, 75]. It was inferred that intact Fgn binding to integrins on endothelial cell surfaces causes conformational change, e.g., exposes the Fgn β^15−42^ domain that directly binds to VE-cadherin [76, 77], translocates to cytosol, and activates MMP9 that disrupts BMEC layer integrity and causes macromolecular leakage due to loss of TJ proteins, such as occludin, ZO-1, and ZO-2 [78, 79]. Our results corroborate these findings, and we observed that increasing the concentration of human plasma Fgn decreased the expression of JAM-A, ZO-1, ZO-2, and VE-cadherin in primary HBMECs.

More recently, mitochondrial dysfunction in BMECs has been associated with BBB dysregulation in neuroinflammatory and neurodegenerative diseases [25, 48, 80–82]. Imbalance activity of fission and fusion leads to mitochondrial dysfunction that affects energy production, apoptosis, mitophagy, mitochondrial movement, and mtDNA stability [83–85]. It has been reported that Fgn induces mitochondrial dysfunction in the skeletal myocytes and causes decreased mitochondrial membrane potential in myotubes [86]. To our knowledge, this is the first time that the deleterious effect of elevated human plasma Fgn on mitochondrial fission/fusion in primary human brain endothelium has been shown. We observed that even normal (2 mg/mL) to slightly high (4 mg/mL) levels of Fgn significantly decreased the expression of pDRP1, FIS1, and OPA1 in primary HBMECs. The DRP1 plays a key role in mitochondrial fission by limiting mitochondria as a mechanochemical GTPase [87, 88]. Complete loss of DRP1 causes embryonic lethality [89, 90], establishing the importance of DRP1 in embryonic development. In addition to genetic mutations in DRP1, varied expressions and anomalous post-translational modifications of DRP1 have been linked to a wide range of age-related neurodegenerative diseases [91–94]. Phosphorylation of DRP1 (pDRP1) by different kinases of different amino acids causes opposite effects. The pDRP1 (Ser616) stimulates mitochondrial fission during mitosis. Conversely, fission is inhibited with pDRP1 Ser637 [95]. In our study, both pDRP1 (Ser616) and pDRP1 (Ser637) were decreased, which indicates that a compensatory mechanism between pDRP1 (Ser616) and pDRP1 (Ser637) has been affected in Fgn-treated primary human brain endothelium. Lin et al. [96] found that only DRP1 was dramatically decreased in the aortic endothelium of old compared with young rats and showed that loss of DRP1 during senescence induces endothelial dysfunction. Our study indicated that increasing the concentration of Fgn significantly decreased pDRP1 in primary HBMECs. Moreover, after stable knockdown of DRP1 by shRNA in primary HBMECs, the expression of BBB-related TJ-proteins (JAM-A and ZO-2) as well as mitochondrial fusion proteins (l-OPA1 and MFN2) was also significantly decreased in DRP1 knockdown cells.

## Limitations and future perspectives of the study

There were several limitations of our study. First, our isolated MVs are mixtures of arterioles, capillaries, and venules. Therefore, we cannot attribute our results to any one segment of the microvasculature. Still, we do not know which techniques would allow us to separate vascular segments with any degree of confidence and still yield enough protein for analysis. Second, because of a large and diverse amount of proteomic data, we could validate only a limited number of proteins by western blotting and immunofluorescence study. Third, the deleterious effect of Fgn on BBB integrity and the increasing permeability was tested in vitro, the in vivo measurement of Fgn-dependent increase of BBB permeability would be a crucial next step. Fourth, similarly, Fgn-mediated dysregulation of mitochondrial fission/fusion with aging also needs to be validated in vivo. Finally, a substantial amount of information provided by our proteomic analysis was excluded from the discussion because of the lack of perceived relevance of the current focus on the dysregulation of BBB and mitochondrial fission/fusion in brain MVs with aging.

## Conclusions

The results of our study support the concept that elevated Fgn could be an early, precipitating event leading to dysregulation in mitochondrial fission/fusion, which subsequently leads to adverse changes in the proteins supporting the integrity of the BBB. Moreover, our results indicate that damaging effects of normal aging occur as early as middle age in mice and thereby offer support for the concept that screening and therapies, especially in individuals vulnerable to cognitive impairment, should begin in mid-life. Our results direct its attention to the need for further investigations, which we expect will lead to novel therapies to protect not only the microvasculature but also the brain parenchyma with aging.

### Supplementary information

Below is the link to the electronic supplementary material.

Supplementary file1 (XLSX 17 KB)

Supplementary file2 (DOCX 20 KB)

Supplementary file3 (DOCX 19 KB)

Supplementary file4 (DOCX 20 KB)

Supplementary file5 (TIF 7968 KB)

## Data Availability

The datasets generated during and/or analyzed during the current study are available from the corresponding author on reasonable request.

## Abbreviations

CNS: Central nervous system
HBMECs: Human brain microvascular endothelial cells
MVs: Brain cortical microvessels
BBB: Blood-brain barrier
TJ: Tight junction
ROS: Reactive oxygen species
IPA: Ingenuity pathway analysis
Fgn: Fibrinogen
FGA: Fibrinogen alpha chain
FGB: Fibrinogen beta chain
FGG: Fibrinogen gamma chain
DRP1: Dynamin-related protein 1
DEPs: Differentially expressed proteins
mtDNA: Mitochondrial DNA
shRNA: Small hairpin RNA
