# Supplementary material for: Fibrinogen in mice cerebral microvessels induces blood–brain barrier dysregulation with aging via a dynamin-related protein 1–dependent pathway
Source: GeroScience. 2023 Oct 28;46(1):395–415. doi: 10.1007/s11357-023-00988-y (PMC10828490; doi:10.1007/s11357-023-00988-y)
Supplement: Supplementary file 2 — Supplementary file2 (DOCX 20 KB) [file 11357_2023_988_MOESM2_ESM.docx]

**Supplementary Table 2: Gene symbol, protein name, and fold change of the proteins presented in Fig. 2B**

| **Permeability of BBB** | | | |
| --- | --- | --- | --- |
| **Symbol** | **Protein Name** | **Fold Change**  **(Middle aged vs Young)** | **Fold Change**  **(Old vs Young)** |
| ANXA1 | annexin A1 | -∞ | 1.509 |
| APP | amyloid beta precursor protein | -1.036 | -1.857 |
| CREB1 | cAMP responsive element binding protein 1 | -∞ | 3.337 |
| DAG1 | dystroglycan 1 | -1.446 | 1.087 |
| ITGB2 | integrin subunit beta 2 | -∞ | -∞ |
| LAMA2 | laminin subunit alpha 2 | -1.153 | -1.298 |
| MERTK | MER proto-oncogene, tyrosine kinase | -4.558 | -2.66 |
| OCLN | occludin | -1.194 | -1.154 |
| PLAT | plasminogen activator, tissue type | -∞ | -2.415 |
| ROCK2 | Rho associated coiled-coil containing protein kinase 2 | -1.351 | -2.445 |
| SLC2A1 | solute carrier family 2 member 1 | -1.113 | 1.195 |
| TYRO3 | TYRO3 protein tyrosine kinase | -1.061 | -1.759 |
| KDR | kinase insert domain receptor | 1.313 | 2.198 |
| MOG | myelin oligodendrocyte glycoprotein | 1.563 | -1.641 |
| SEMA7A | semaphorin 7A (John Milton Hagen blood group) | 1.455 | -1.177 |
| CREBBP | CREB binding protein | N.Q. | ∞ |
| **Leakage of BBB** | | |  |
| PLAT | plasminogen activator, tissue type | -∞ | -2.415 |
| SERPINI1 | serpin family I member 1 | -1.33 | -3.567 |
| **Damage of BBB** | | |  |
| DTNA | dystrobrevin alpha | -1.834 | -1.339 |
| IKBKB | inhibitor of nuclear factor kappa B kinase subunit beta | -∞ | -∞ |
| IGHM | immunoglobulin heavy constant mu | 2.653 | 2.8 |
| **Breakdown of BBB** | | | |
| LRP1 | LDL receptor related protein 1 | -1.392 | -1.59 |
| IL33 | interleukin 33 | 1.171 | 2.465 |
| **Integrity of BBB** | | | |
| OCLN | occludin | -1.194 | -1.154 |
| PRKAA1 | protein kinase AMP-activated catalytic subunit alpha 1 | 1.955 | 3.544 |
| SOD1 | superoxide dismutase 1 | 1.224 | -2.436 |
| SOD2 | superoxide dismutase 2 | 1.086 | -1.658 |
| IGHM | immunoglobulin heavy constant mu | 2.653 | 2.8 |
| **Physiological function of BBB** | | | |
| AQP4 | aquaporin 4 | -1.752 | -1.203 |
| NOS1 | nitric oxide synthase 1 | -2.158 | -∞ |
| NOS3 | nitric oxide synthase 3 | -1.524 | -1.235 |
| NR3C1 | nuclear receptor subfamily 3 group C member 1 | -7.704 | 1.425 |
| OCLN | occludin | -1.194 | -1.154 |
| PRKAA1 | protein kinase AMP-activated catalytic subunit alpha 1 | 1.955 | 3.544 |
| SOD1 | superoxide dismutase 1 | 1.224 | -2.436 |
| SOD2 | superoxide dismutase 2 | 1.086 | -1.658 |
| CLDN5 | claudin 5 | 1.127 | 2.654 |
| GFAP | glial fibrillary acidic protein | 1.517 | 1.454 |
| IGHM | immunoglobulin heavy constant mu | 2.653 | 2.8 |
|  |  |  |  |
