# Supplementary material for: Fibrinogen in mice cerebral microvessels induces blood–brain barrier dysregulation with aging via a dynamin-related protein 1–dependent pathway
Source: GeroScience. 2023 Oct 28;46(1):395–415. doi: 10.1007/s11357-023-00988-y (PMC10828490; doi:10.1007/s11357-023-00988-y)
Supplement: Supplementary file 3 — Supplementary file3 (DOCX 19 KB) [file 11357_2023_988_MOESM3_ESM.docx]

**Supplementary Table 3: Name, location, and the fold change of the proteins**

**presented in Figure 4**

| **Symbol** | **Protein Name** | **Location** | **Fold Change** |
| --- | --- | --- | --- |
| CTNNA1 | catenin alpha 1 | Plasma Membrane | -1.413 |
| CTNNB1 | catenin beta 1 | Nucleus | -3.039 |
| CLDN11 | claudin 11 | Plasma Membrane | -1.211 |
| EPB41 | erythrocyte membrane protein band 4.1 | Plasma Membrane | -1.826 |
| FGA | fibrinogen alpha chain | Extracellular Space | 1.202 |
| FGB | fibrinogen beta chain | Extracellular Space | 1.236 |
| FGG | fibrinogen gamma chain | Extracellular Space | 1.001 |
| JAM3 | junctional adhesion molecule 3 | Plasma Membrane | -1.224 |
| MYH9 | myosin heavy chain 9 | Cytoplasm | -1.067 |
| MYH10 | myosin heavy chain 10 | Cytoplasm | -1.902 |
| MYH14 | myosin heavy chain 14 | Extracellular Space | -1.357 |
| MYL6 | myosin light chain 6 | Cytoplasm | -2.932 |
| MYL9 | myosin light chain 9 | Cytoplasm | -1.254 |
| MYO18A | myosin XVIIIA | Cytoplasm | -1.546 |
| NECTIN1 | nectin cell adhesion molecule 1 | Plasma Membrane | -∞ |
| NECTIN3 | nectin cell adhesion molecule 3 | Plasma Membrane | -∞ |
| NAPA | NSF attachment protein alpha | Cytoplasm | -1.388 |
| NAPB | NSF attachment protein beta | Cytoplasm | -1.532 |
| NAPG | NSF attachment protein gamma | Cytoplasm | -1.889 |
| OCLN | occludin | Plasma Membrane | -1.154 |
| PLAT | plasminogen activator, tissue type | Extracellular Space | -2.415 |
| PRKACA | protein kinase cAMP-activated catalytic subunit alpha | Cytoplasm | -1.384 |
| PRKACB | protein kinase cAMP-activated catalytic subunit beta | Cytoplasm | -1.309 |
| PRKAR2B | protein kinase cAMP-dependent type II regulatory subunit bet | Cytoplasm | -1.705 |
| PPP2CA | protein phosphatase 2 catalytic subunit alpha | Cytoplasm | -1.652 |
| RAC1 | Rac family small GTPase 1 | Plasma Membrane | -1.751 |
| RHOA | ras homolog family member A | Cytoplasm | -1.459 |
| SNAP25* | synaptosome associated protein 25 | Plasma Membrane | -1.482 |
| STX4 | syntaxin 4 | Plasma Membrane | -2.613 |
| STX16 | syntaxin 16 | Cytoplasm | -2.619 |
| STX1A | syntaxin 1A | Cytoplasm | -1.780 |
| STX1B | syntaxin 1B | Plasma Membrane | -2.462 |
| ZO1 | tight junction protein 1/ zonula occludens 1 | Plasma Membrane | -2.001 |
| ZO2 | tight junction protein 2/ zonula occludens 2 | Plasma Membrane | -1.359 |
