# Supplementary material for: Fibrinogen in mice cerebral microvessels induces blood–brain barrier dysregulation with aging via a dynamin-related protein 1–dependent pathway
Source: GeroScience. 2023 Oct 28;46(1):395–415. doi: 10.1007/s11357-023-00988-y (PMC10828490; doi:10.1007/s11357-023-00988-y)
Supplement: Supplementary file 4 — Supplementary file4 (DOCX 20 KB) [file 11357_2023_988_MOESM4_ESM.docx]

**Supplementary Table 4: Name, location, and the fold change of the proteins presented in Figure 5**

| **Symbol** | **Protein Name** | **Location** | **Fold Change** |
| --- | --- | --- | --- |
| ACACA | acetyl-CoA carboxylase alpha | Cytoplasm | -1.805 |
| AFG3L2 | AFG3 like matrix AAA peptidase subunit 2 | Cytoplasm | -2.894 |
| BAX | BCL2 associated X, apoptosis regulator | Cytoplasm | -∞ |
| CLPP | caseinolytic mitochondrial matrix peptidase proteolytic subunit | Cytoplasm | -1.270 |
| CHCHD3 | coiled-coil-helix-coiled-coil-helix domain containing 3 | Cytoplasm | -3.775 |
| DDHD1 | DDHD domain containing 1 | Extracellular Space | -∞ |
| DAP3 | death associated protein 3 | Cytoplasm | -2.114 |
| DHODH | dihydroorotate dehydrogenase (quinone) | Cytoplasm | -1.210 |
| DRP1 | dynamin related protein 1 | Cytoplasm | -2.205 |
| FGA | fibrinogen alpha chain | Extracellular Space | 1.202 |
| FGB | fibrinogen beta chain | Extracellular Space | 1.236 |
| FGG | fibrinogen gamma chain | Extracellular Space | 1.001 |
| FIS1 | fission, mitochondrial 1 | Cytoplasm | -1.501 |
| GDAP1 | ganglioside induced differentiation associated protein 1 | Cytoplasm | -1.347 |
| GIPC1 | GIPC PDZ domain containing family member 1 | Cytoplasm | -2.260 |
| LRP1 | LDL receptor related protein 1 | Plasma Membrane | -1.590 |
| MARCHF5 | membrane associated ring-CH-type finger 5 | Cytoplasm | -1.898 |
| MAPT* | microtubule associated protein tau | Plasma Membrane | ∞ |
| MCU | mitochondrial calcium uniporter | Cytoplasm | -1.436 |
| MUL1 | mitochondrial E3 ubiquitin protein ligase 1 | Cytoplasm | -∞ |
| Mff* | mitochondrial fission factor | Cytoplasm | -1.952 |
| MTFP1 | mitochondrial fission process 1 | Cytoplasm | -1.864 |
| MFN1 | mitofusin 1 | Cytoplasm | -1.001 |
| MFN2 | mitofusin 2 | Cytoplasm | -1.679 |
| MIGA1 | mitoguardin 1 | Plasma Membrane | -∞ |
| MIGA2 | mitoguardin 2 | Plasma Membrane | -1.280 |
| OPA1 | OPA1 mitochondrial dynamin like GTPase | Cytoplasm | -2.046 |
| PARL | presenilin associated rhomboid like | Cytoplasm | -1.200 |
| PTPMT1 | protein tyrosine phosphatase mitochondrial 1 | Cytoplasm | -1.307 |
| SLC25A46 | solute carrier family 25 member 46 | Cytoplasm | -4.310 |
| STOML2 | stomatin like 2 | Plasma Membrane | -1.562 |
| SNCA | synuclein alpha | Cytoplasm | -2.753 |
| UBE2N | ubiquitin conjugating enzyme E2 N | Cytoplasm | -9.169 |
| VPS35 | VPS35 retromer complex component | Cytoplasm | -3.34 |
