# Supplementary figures and images for: Fibrinogen in mice cerebral microvessels induces blood–brain barrier dysregulation with aging via a dynamin-related protein 1–dependent pathway
Source: GeroScience. 2023 Oct 28;46(1):395–415. doi: 10.1007/s11357-023-00988-y (PMC10828490; doi:10.1007/s11357-023-00988-y)

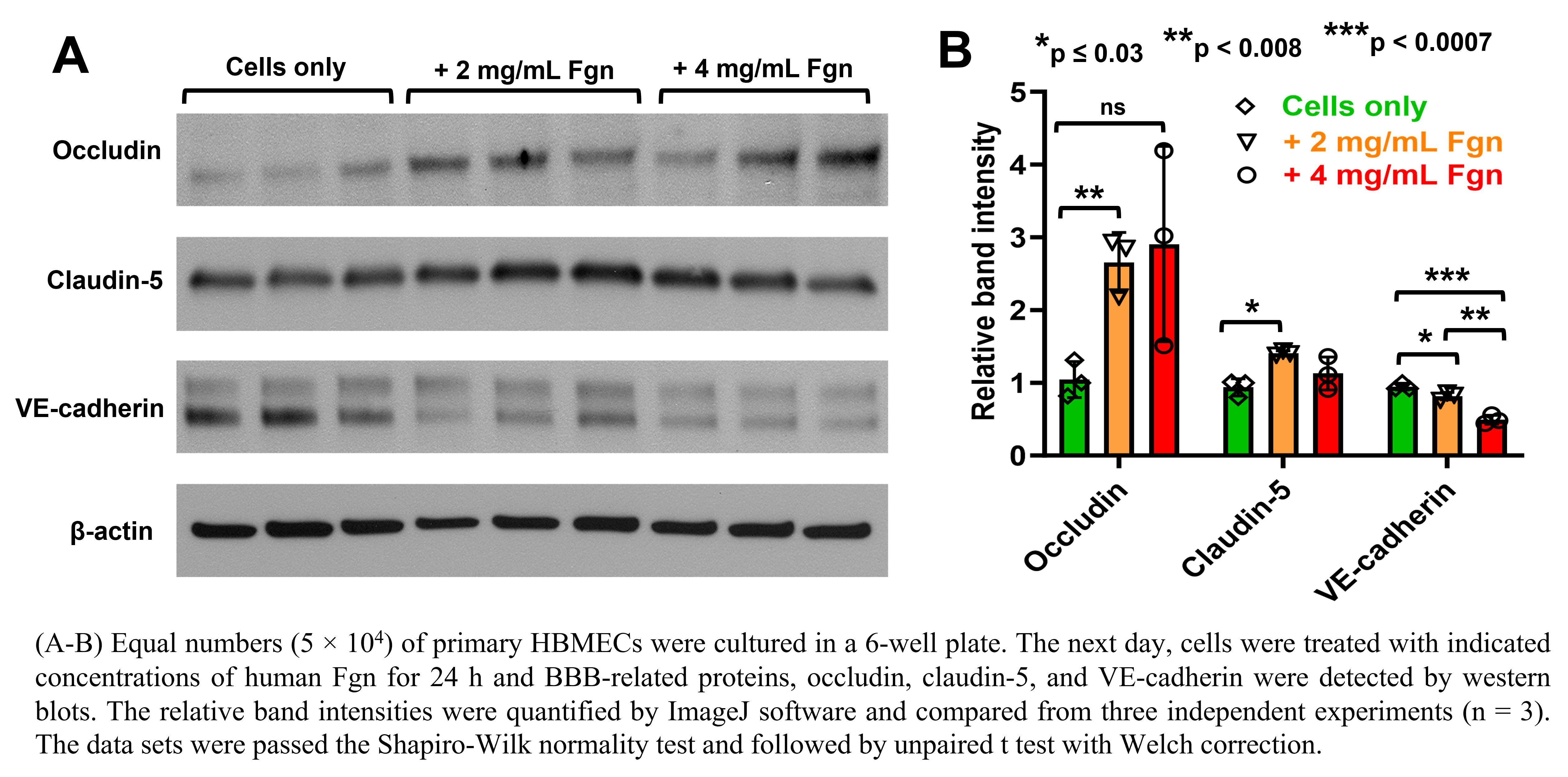

Supplement: Supplementary file 5 — Supplementary file5 (TIF 7968 KB) [file 11357_2023_988_MOESM5_ESM.tif]
